# Genetic Variation in the *Vascular Endothelial Growth Factor (VEGFA*) Gene at rs13207351 Is Associated with Overall Survival of Patients with Head and Neck Cancer

**DOI:** 10.3390/cancers13051163

**Published:** 2021-03-08

**Authors:** Foteinos-Ioannis Dimitrakopoulos, Georgia-Angeliki Koliou, Vassiliki Kotoula, Kyriaki Papadopoulou, Konstantinos Markou, Konstantinos Vlachtsis, Nikolaos Angouridakis, Ilias Karasmanis, Angelos Nikolaou, Amanda Psyrri, Anastasios Visvikis, Paris Kosmidis, George Fountzilas, Angelos Koutras

**Affiliations:** 1 Division of Oncology & Molecular Oncology Laboratory, Department of Medicine, University Hospital, Medical School, University of Patras, 26504 Patras, Greece; fodimitrakopoulos@upatras.gr; 2 Section of Biostatistics, Hellenic Cooperative Oncology Group, Data Office, 11526 Athens, Greece; g_koliou@hecog.ondsl.gr; 3 Department of Pathology, School of Health Sciences, Faculty of Medicine, Aristotle University of Thessaloniki, 54006 Thessaloniki, Greece; vkotoula@auth.gr; 4 Laboratory of Molecular Oncology, Hellenic Foundation for Cancer Research/Aristotle University of Thessaloniki, 54006 Thessaloniki, Greece; k_papadopoulou@hecog.ondsl.gr (K.P.); fountzil@auth.gr (G.F.); 5 Second Academic Otorhinolaryngology-Head and Neck Surgery Department, Papageorgiou Hospital, Aristotle University of Thessaloniki, 56429 Thessaloníki, Greece; kmarkou@med.auth.gr (K.M.); orl2@med.auth.gr (N.A.); 6 Otolaryngology Department, G. Papanikolaou General Hospital, 57010 Thessaloniki, Greece; vlachtsisk@gpapanikolaou.gr (K.V.); dorl@ippokratio.gr (I.K.); nikolaoua@gpapanikolaou.gr (A.N.); 7 Department of Internal Medicine, Section of Medical Oncology, Attikon University Hospital, National and Kapodistrian University of Athens, 12462 Athens, Greece; dpsyrri@med.uoa.gr; 8 Third Department of Medical Oncology, Agii Anargiri Cancer Hospital, 14564 Athens, Greece, hecogaga@otenet.gr; 9 Second Department of Medical Oncology, Hygeia Hospital, 15123 Athens, Greece; p.kosmidi@hygeia.gr; 10 Department of Medical Oncology, German Oncology Center, 4108 Limassol, Cyprus

**Keywords:** head and neck cancer, nasopharyngeal cancer, VEGFA, FAS, endothelin, NBS1, SNPs

## Abstract

**Simple Summary:**

Angiogenesis and apoptosis play a pivotal role in the pathogenesis and clinical course not only of nasopharyngeal cancer (NPC), but also of other subgroups of head and neck cancer (HNC), such as laryngeal cancer. Thus, the aim of this study was to investigate the clinical significance of genetic polymorphisms in four pivotal angiogenesis- and apoptosis-related genes (*VEGFA*, *FAS*, *EDNRA* and *NBS1*) in HNC patients. Thirty-four genetic variants located in the studied genes were assessed. Two of them (*VEGFA* rs13207351 and *FAS* rs2234768) were associated with overall survival for patients with laryngeal cancer and NPC, respectively, with *VEGFA* rs13207351 showing the most promise for its prognostic value in the subgroup of laryngeal cancer patients. This study suggests that genetic variations in angiogenesis- and apoptosis-related genes may be useful in the management of HNC patients.

**Abstract:**

Head and neck cancer (HNC) is a significantly heterogeneous disease and includes malignancies arising from different anatomical sites, such as nasopharyngeal cancer (NPC) and laryngeal cancer (LC). In the current study, polymorphisms located in angiogenesis- and apoptosis-related genes (*VEGFA*, *FAS*, *EDNRA* and *NBS1*) were evaluated regarding their clinical significance in HNC patients. In total, 333 HNC patients were enrolled in this study and 34 variants located on the aforementioned genes were genotyped via Sanger sequencing. LC patients, homozygous A for *VEGFA* rs13207351, had shorter overall survival (OS) as opposed to homozygous G (Hazard ratio (HR) = 2.06, Wald’s *p =* 0.017) upon adjustment for age, disease stage, and surgery. Following the dominant model, LC patients carrying the A allele had a marginally significantly higher risk for death (HR = 1.72, *p* = 0.059). NPC patients heterozygous (CT) for *FAS* rs2234768 had a marginal but significantly higher risk of death compared to those with homozygosity for the T allele (HR = 2.22, *p* = 0.056). In conclusion, rs13207351 (*VEGFA*) and rs2234768 (*FAS*) polymorphisms seem to have prognostic significance in HNC, with *VEGFA* rs13207351 showing the most promise in this subgroup of LC patients.

## 1. Introduction

Head and neck cancer (HNC) is the seventh most frequent cancer type worldwide [1]. According to Global Cancer Observatory (GLOBOCAN) 2020, cancers of the lips, oral cavity, larynx, and pharynx account for 4.6% of all new cases and deaths globally [2]. In the USA, the estimated new cases and deaths due to malignancies in the oral cavity, pharynx and larynx during the current year will be 66,630 and 14,620, respectively [3]. In Europe, approximately 140,000 cases and 63,500 deaths due to HNC were reported in 2012 [4]. HNC is associated with an increased consumption of alcohol and/or the use of tobacco [5], whereas some specific subtypes are closely related to human papillomavirus (HPV) [6] and Epstein–Barr virus (EBV) infection [7].

HNC is characterized by significant heterogeneity, as it includes cancers arising from different anatomical sites in the head and neck. In addition, this group of carcinomas is also heterogeneous regarding the molecular mechanisms involved in the pathophysiology of the disease, as well as their pathological characteristics and their embryological origin. The majority (more than 90%) of HNCs present with squamous cell histology [8]. Nasopharyngeal carcinoma (NPC), a type of HNC, also has squamous histology and is categorized into three histological subtypes according to World Health Organization (WHO) classification: keratinizing (20–25%), non-keratinizing undifferentiated type (60–65%), and non-keratinizing differentiated type (10–15%) [9]. NPC mainly develops in the nasopharyngeal portion of the pharynx, which is derived from the neural crest of the ectodermal leaflet, while laryngeal cancer (LC), the main non-NPC carcinoma in the current study, is derived from the larynx, which arises from the 4th and 6th pharyngeal arches in combination with an endodermal inner surface lining [10,11]. NPC is rare in Caucasians from Western countries, with the annual incidence ranging between 0.1–1.1/100,000 people, while it reaches 1/100,000 inhabitants in Southeast Europe [12,13]. In contrast, NPC is deemed to be endemic in China, Southeast Asia, and Africa [14].

The role of EBV infection in NPC pathogenesis and progression is well documented, and due to this direct etiological implication, NPC has been characterized as a virus-related malignancy [7]. In addition, EBV infection has also been associated with the undifferentiated histological subtype of NPC, which is the most common subtype in endemic countries. In the USA and North European countries, differentiated and non-keratinizing squamous cell carcinoma is the predominant histology of NPC [15].

A pivotal cancer hallmark, aberrant in NPC as well as in HNC, is angiogenesis [16,17]. One of the most essential angiogenesis-related molecules is vascular endothelial growth factor A (VEGFA), which has attracted the interest of the scientific community regarding its role in both NPC and HNC [18]. Amongst the most interesting issues about angiogenesis is the clinical value of genetic variations in the *VEGFA* gene, a topic that has been studied in many cancer types from our as well as other research groups [19,20,21,22,23,24].

Furthermore, the endothelin-1 (END1)/endothelin A receptor (EDNRA) axis also plays an essential role in angiogenesis [25]. In particular, EDNRA has been reported as an independent prognostic value for distant metastases in patients with NPC, while almost three-quarters of NPCs overexpress EDNRA [26]. Similar to *VEGFA*, variants of *EDNRA* have also been associated with the prognosis of patients with locoregionally advanced NPC [27], as well as with reduced post radiotherapy xerostomia [28].

A significant role has also been attributed to apoptosis in HNC, which is particularly deregulated, leading to the bypass of apoptotic signals. One of the major regulators of apoptosis is the Fas cell surface death receptor (FAS) protein, a cell surface receptor that interacts with FAS ligand (FASL), leading to apoptosis [29]. Genetic variants of the *FAS* gene have been documented to interrupt apoptosis-related signal transduction pathways, destabilizing the balance between cell death and proliferation [30]. In particular, a meta-analysis of 52 studies by Xu et al., showed that genetic variations of *FAS* are associated with a decreased or increased risk of different types of cancer [31]. Additionally, *FAS* rs1800682 (-670A>G) has been associated with susceptibility to prostate cancer, esophageal cancer, hepatocellular carcinoma [32], and papillary thyroid cancer [33], but not with cervical cancer [34].

Furthermore, defects in DNA repair mechanisms have also been observed in HNC [35]. A central molecule in cellular response to double-strand DNA breaks is nibrin (NBS1), which participates in the MRN complex (MRE11(Meiotic Recombination 11 Homolog 1)/RAD50 (RAD50 Double Strand Break Repair Protein)/NBS1(Nibrin)), promoting DNA repair and chromosome integrity [36,37]. Genetic polymorphisms in the *NBS1* gene may impair the mechanism of responding to DNA damage, creating a predisposition for cancer, whereas mutations in the same gene have been related to Nijmegen breakage syndrome (NBS) [38].

In this study, we evaluated the clinical significance of 34 genetic polymorphisms located in the *VEGFA, EDNRA, FAS* and *NBS1* genes in patients with confirmed NPC or non-NPC HNC.

## 2. Results

### 2.1. Patient Characteristics

Among the 333 patients included in this study, 144 (43.2%) had NPC and the rest (56.8%) were diagnosed with non-NPC disease. The majority of the patients with non-NPC had squamous cell carcinomas (96.6%), tumors located in the larynx (85.6%), and had undergone surgery (81.3%). More than half of the NPC patients had undifferentiated carcinomas (58.1%) and were mostly stage III (38.6%). Selected patient and tumor characteristics for the entire cohort are presented in Table 1 by tumor type.

### 2.2. Variant Distribution

The frequency distribution of the examined variants for the entire cohort and separately for patients with NPC and non-NPC is presented in Table 2. In the non-NPC group of patients, analysis focused on patients with tumors located in the larynx (*n* = 149), thus leading to a total of 293 patients. Genotyping of *VEGFA* polymorphisms revealed that all patients were homozygous G for rs12664104 and rs112005313, homozygous T for rs34376996 and rs111933757, homozygous A for rs28357093 and for rs187429037, and homozygous C for rs79469752, rs59260042, rs149179279 and rs112256643. All non-NPC patients were homozygous C for rs149983590, whereas only one of the NPC patients (0.7%) had a heterozygous CA genotype. The observed genotypes for the aforementioned *VEGFA* single nucleotide polymorphisms (SNPs) in our study, including rs833062, are expected for low frequency or rare SNPs and are in accordance with the respective minor allele frequency (MAF) values in the global and/or European population, as reported in the Single Nucleotide Polymorphism Database (dbSNP) database according to 1000 Genomes Project (1000G), Exome Aggregation Consortium (ExAC), Genome Aggregation Database (gnomAD) or Allele Frequency Aggregator project (ALFA) projects (https://www.ncbi.nlm.nih.gov/snp/, accessed on 10 October 2020). In the case of common *VEGFA* polymorphisms, most patients were heterozygous AC for rs699947 (49% of non-NPC and 46% of NPC patients), heterozygous for the rs144854329 18 bp indel (49% of non-NPC and 53.6% of NPC), heterozygous for the 1 bp insertion rs35864111 (49% of non-NPC and 53.6% of NPC) and heterozygous CT for rs833061 (referred to as −1498 herein) (50.3% for non-NPC and 48.8% for NPC). These findings are in line with previous haplotype analysis studies, at least for rs699947 and rs144854329 or −1498, which showed strong linkage disequilibrium among the two polymorphisms in both cases [20,39,40]. Additionally, both non-NPC and NPC patients were mostly homozygous A for rs13207351 (46.3% for non-NPC and 47.9% for NPC) and homozygous C for +936 (70.3% for non-NPC and 70.1% for NPC). Finally, most non-NPC patients were homozygous A for −1154 (40.3%), whereas most NPC patients were homozygous G for −1154 (43.6%). The observed frequencies of the common *VEGFA* polymorphisms described above are comparable to the minor allele frequencies (MAFs) reported for the global and/or European population in dbSNP. The genotyping of *EDNRA* variants revealed that none of the patients harbored p.L322V (all were homozygous C). Most of the patients were homozygous T for p.H323H (56.8% of non-NPC and 55.3% of NPC patients) and homozygous G for p.E335E (56.8% of non-NPC and 56.1% of NPC patients), consistent with the reported dbSNP MAFs for these common SNPs. Concerning rare *EDNRA* rs10305924 and rs112710542 SNPs, all non-NPC patients were homozygous G for both, whereas only one NPC patient (0.7%) was heterozygous AG for rs10305924 and six NPC patients (4.3%) were heterozygous AG for rs112710542. For *FAS,* more than half of the patients were heterozygous AG for −670 among both NPC (63.8%) and non-NPC patients (50.3%). The majority of non-NPC (81.2%) and NPC patients (82.3%) were homozygous T for −690, whereas all patients were homozygous T for rs34995925 and homozygous A for rs150130637. In all instances, the observed genotype frequencies for *FAS* promoter SNPs in our study were in agreement with their reported MAFs in National Center for Biotechnology Information (NCBI) dbSNP.

Regarding *NBS1* rare SNPs, all patients were homozygous T for rs192240705 and homozygous A for p.A183A. None of the patients carried p.A183T (homozygous G) or p.R169C (homozygous C), whereas only two patients (0.7%) were heterozygous AG for p.I171V—one with non-NPC and one with NPC. Finally, GC heterozygosity was observed in most non-NPC (45%) and NPC (54.9%) patients for *NBS1* p.E185Q.

### 2.3. Association of Variants with Clinicopathological Characteristics

**Figure 1:**
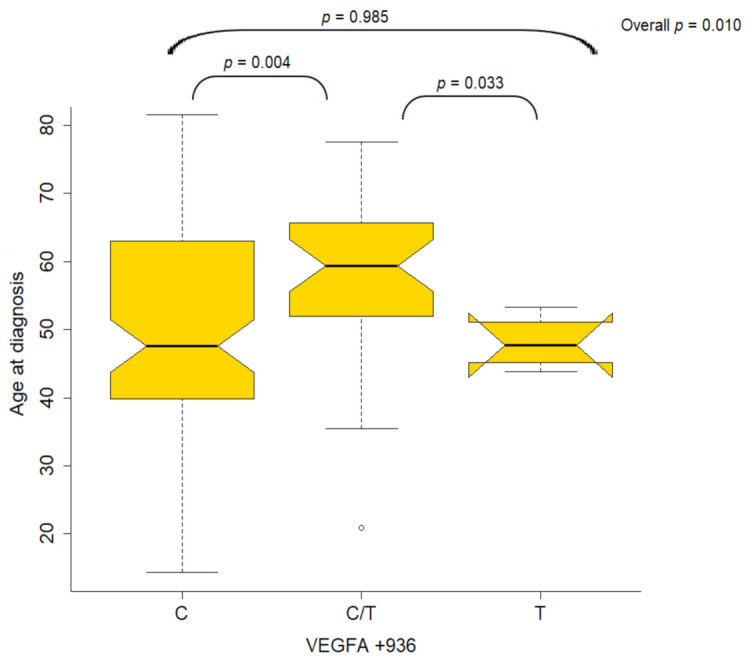
Boxplots of the distribution of age for VEGFA +936 genotypes in NPC patients.

A significant association was found between *VEGFA* +936 and age at diagnosis (Wilcoxon rank-sum *p* = 0.010) in NPC patients. NPC patients who were heterozygous CT for *VEGFA* +936 were diagnosed at an older age compared to those who were homozygous T (median age 59.4 vs. 47.7 years, Wilcoxon rank-sum *p* = 0.033) and patients who were homozygous C (median age 59.4 vs. 47.6, Wilcoxon rank-sum *p* = 0.004) (Figure 1). Among non-NPC patients, FAS rs2234768 (−690) was significantly associated with alcohol abuse (Fisher’s *p* = 0.033). More specifically, alcohol abuse was more frequent in patients with homozygous T FAS rs2234768 (−690) compared to those with heterozygous CT (81% vs. 59.3%).

### 2.4. Improved Overall Survival for NPC Patients

Survival data were available for 272 of 293 patients (92.8%); overall, 148/149 LC (99.3%) and 124 of 144 NPC patients (86.1%). At a median follow-up of 119.4 months [95% confidence interval (CI): 70–130.5], 32 NPC patients had died (25.8%), while the median OS had not been reached at the time of analysis. Patients with LC were followed-up for a median of 95.6 months (95% CI: 74.0–124.8), and during this period, 71 deaths (48%) were reported in LC patients. The median OS for LC patients was 88.6 months (95% CI: 62.2–135.5).

### 2.5. FAS −690 and Overall Survival in NPC Patients

**Figure 2:**
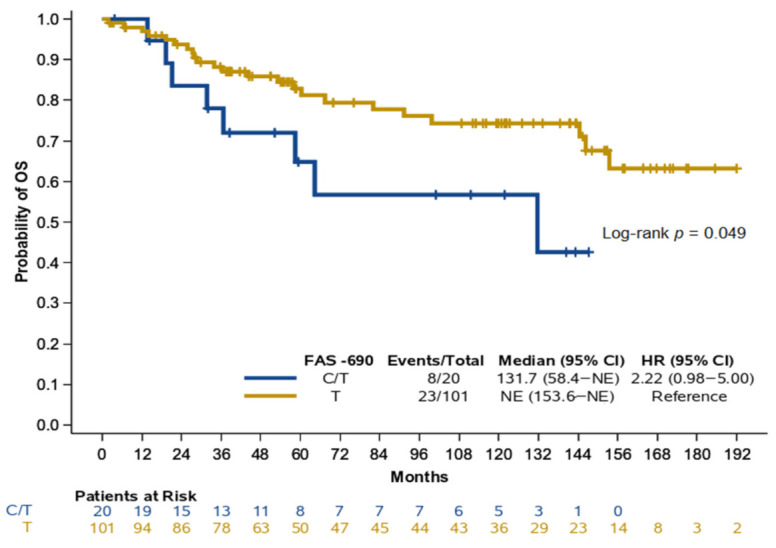
Kaplan–Meier curves for OS according to FAS −690 genotypes in NPC patients. HR: hazard ratio, CI: confidence interval, NE: not estimable.

NPC patients with heterozygous CT for *FAS* −690 were at a marginal but significantly higher risk of death compared to those with homozygous T (Hazard Ratio (HR) = 2.22, 95% CI: 0.98–5.00, Wald’s *p* = 0.056, Figure 2); the rest of the variants were not found to be prognostic for OS among patients with NPC (Table S1). Upon adjustment for age and stage, *FAS* −690 was not found to be a significant prognosticator for OS (*p* = 0.66) among NPC patients, even though the hazard ratio for patients with heterozygous CT was in the same direction as the one obtained by the univariate analysis (HR = 1.23, 95% CI: 0.48–3.17). We further assessed the potential prognostic significance of variants among NPC patients treated with primary concomitant chemotherapy and radiotherapy (*N* = 114) since this subgroup accounted for the vast majority of NPC patients. As in the entire cohort of NPC patients, heterozygous CT for *FAS* −690 as compared to homozygous T showed a marginal but significantly higher risk of death univariately in the subpopulation of NPC patients treated with primary concomitant chemotherapy and radiotherapy (HR = 2.27, 95% CI: 1.00–5.18, *p* = 0.051), which was not retained upon adjustment for age and stage (HR = 1.32, 95% CI: 0.50–3.44, *p* = 0.58).

### 2.6. LC Patients Carrying the A Allele of VEGFA rs13207351 Had a Higher Risk of Death and Poorer Outcomes

**Figure 3:**
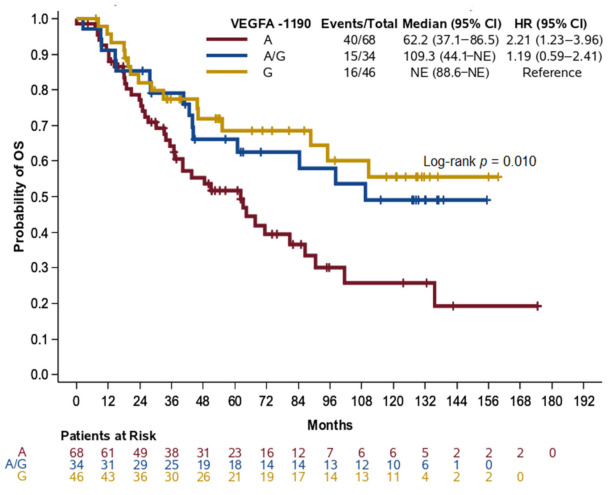
Kaplan–Meier curves for OS according to VEGFA −1190 genotypes among non-NPC patients. HR: hazard ratio, CI: confidence interval, NE: not estimable.

LC patients with homozygous A for *VEGFA* rs13207351 (referred to as −1190 herein) had shorter OS compared to LC patients with homozygous G (HR = 2.21, 95% CI: 1.23–3.96, *p* = 0.008, Figure 3). Homozygosity A for *VEGFA* −1190 retained its unfavorable prognostic significance for OS over homozygosity G in LC patients (HR = 2.06, 95% CI: 1.14–3.72, *p =* 0.017) after adjustment for age (*p =* 0.69), disease stage (*p =* 0.10), and surgery (*p =* 0.87), thus underlining the independent prognostic significance of *VEGFA* −1190. We further examined the dominant and recessive genetic models for *VEGFA* −1190 in LC patients. Assuming that A is the risk allele, the dominant genetic model confirmed that carriers of the *VEGFA* −1190 A allele (A+AG) had a higher risk of death compared to homozygotes for the G allele univariately (HR = 1.79, 95% CI: 1.02–3.12, *p =* 0.042), while marginal significance was retained after adjustment for age, stage, and surgery (HR = 1.72, 95% CI: 0.98–3.03, *p =* 0.059, Table 3).

## 3. Discussion

Despite the advances in the management of HNC during the last few decades, the survival outcome of patients with HNC remains very poor, with the exception of some specific subgroups that are characterized by better clinical course [1]. HNC is a heterogeneous disease not only regarding the clinical manifestations and course, but also in the genetic variations present [41]. Therefore, the study of these genetic variations not only on sheds light on the underlying mechanisms, but also uncovers clinically useful biomarkers, which can contribute to the personalization of treatment. In the present study, 34 variants in the *VEGFA*, *EDNRA*, *NBS1* and *FAS* genes were assessed in HNC patients (NPC vs. non-NPC), and the results from genotyping were correlated with clinical and pathological characteristics, as well as with survival outcome. The most interesting finding in our study was the association of the common *VEGFA* rs13207351 (−1190) SNP with survival outcome. In LC patients, carriers of the A allele had an increased risk of death during the observation period and poorer outcome. This SNP, which is located in the promoter region of the *VEGFA* gene in a region marked by H3K27Ac and H3K4me3, has a high possibility of being a transcription factor binding site (Figure S1) [42,43] and has been extensively studied in the context of diabetes mellitus. For instance, homozygotes for the minor allele of this SNP have been associated with an increased risk of developing diabetic retinopathy in Chinese patients with type 2 diabetes mellitus [44], but not with the risk for diabetic foot ulcers in the Chinese Han population [45]. Limited data regarding the possible role of rs13207351 in cancer come from a study by our group in patients with metastatic breast cancer that were treated with weekly docetaxel. In that study, homozygotes for the rs13207351 G allele had shorter Progression-free Survival (PFS) compared to carriers of the A allele (combined patients with AG and AA) [46]. Although these studies did not have the same endpoint, as the current study evaluated the prognostic value whereas the previous study evaluated the predictive value of this SNP, this difference may reflect the distinct molecular backgrounds of metastatic breast cancer and HNC, as well as the differences in management and the systematic regimens administered.

Our study further revealed an association of the *FAS* −690 SNP with OS in NPC patients. The clinical significance of this SNP has not been studied in cancer. However, this SNP is located in a regulatory genomic region (positive strand) marked by H3K27Ac and H3K4me3, and has a high possibility of being a transcription factor binding site (Figure S2) [42,43]. Its location at the promoter of the *FAS* gene, combined with the significance of the same gene in NPC, leads us to speculate that this SNP may influence underlying pathophysiological mechanisms. This notion is further supported by the findings of Ho et al., who have shown that Fas ligand expression in NPC patients was associated with poorer disease-free survival, as well as poorer overall survival [47]. In addition, other SNPs close to *FAS* −690 SNP have also been implicated in the clinical outcome of patients with NPC. Bel Hadj Jrad et al., have shown that G allele carriers of the *FAS* −670 A>G SNP had a significantly increased risk for NPC [48]. Additionally, the G allele of the same SNP has been associated with an increased risk for lymph node infiltration, as well as distant metastases, in a cohort of Chinese patients with NPC [49]. Two other functional SNPs of the FAS/FASL axis (*FAS* −1377G/A and *FASL* −844T/C) have also been associated with the development of NPC [50]. Interestingly, the *FAS* −1377 A allele changes the binding site of the Sp1 transcription factor, decreasing *FAS* gene expression [51,52]. Thus, further evaluation in a larger cohort of patients is needed to clarify the potential role of this SNP in NPC.

We have to acknowledge some limitations in the current study. First, a major limitation of our study is the number of included patients, which did not permit potent significant associations to be uncovered. Moreover, the heterogeneity of cohort regarding histological identity, as well as treatment management, are limitations of this study. Furthermore, a two-phase design would have been more informative, providing vali-dated results. Assessment of the same SNPs in healthy controls could provide more information regarding their value as risk factors for the development of HNC. 

In conclusion, two SNPs in the promoter region of the *FAS* (rs2234768) and *VEGFA* (rs13207351) genes seem to be associated with the clinical outcome of patients with HNC, with *VEGFA* rs13207351 being the most promising. Although these findings need further validation in larger cohorts of NPC and LC patients, this study suggests that genetic variations in angiogenesis- and apoptosis-related genes may be useful in the management of HNC patients.

## 4. Patients and Methods

### 4.1. Study Design, Population and Data Collection

The present study was performed upon the approval by the Bioethics Committee of the Aristotle University of Thessaloniki, School of Health Sciences, Faculty of Medicine (#2/23 March 2016) following the Helsinki Declaration (2013) on ethical guidelines [53].

Written informed consent was obtained from all participants unless the Committee had granted a waiver. Patients with histologically confirmed HNC, exclusively of Greek origin, were retrieved from the electronic database of the Hellenic Cooperative Oncology Group (HeCOG). Available germline DNA samples for patients under study were retrieved from the HeCOG Tumor Repository. Genotyping was performed in the Laboratory of Molecular Oncology (MOL) by the Hellenic Foundation for Cancer Research/Aristotle University of Thessaloniki, Thessaloniki, Greece.

### 4.2. Genetic Variant Selection

We selected known *VEGFA, EDNRA, FAS* and *NBS1* SNPs relevant to nasopharyngeal and other cancers based on previous studies by our group and others, as well as on adequate MAF data and a high rate of heterozygosity in the general population according to dbSNP, 1000G, ExAC and GnomAD databases [19,20,21,23,31,39,46,54,55,56,57,58]. Notably, our genotype analysis by Sanger sequencing allowed us to examine nearby genetic variations in addition to each interrogated SNP, resulting in the examination of 34 variants in the four genes [46]. 

For *VEGFA,* we assessed 19 polymorphisms, with 13 located within the 5′ regulatory promoter region, including six common polymorphisms, four SNPs (rs699947 (known as −2578C>A), rs833061 (−1498 or −460C>T), rs13207351 (−1190 or −152G>A), and rs1570360 (known as −1154G>A)), two indels (rs144854329 (-2549) and rs35864111)), and seven SNPs with low (rs34376996, rs833062 (−1455T>C), rs59260042 (−1210 or −172C>A), rs79469752 (−1203 or −165C>T), rs28357093 (−1179 or −141A>C)) or rare (rs149983590 and rs12664104) frequencies. The other six *VEGFA* polymorphisms resided within the 3′ untranslated region and represented one common (rs3025039 (known as +936C>T)) and five rare (rs111933757, rs187429037, rs112005313, rs112256643, and rs149179279) SNPs. For *FAS*, we analyzed two common (rs1800682 (known as −670A>G) and rs2234768 (known as −690T>C)) and two rare (rs34995925 (−663T>C) and rs150130637) SNPs in the promoter region. In the case of *EDNRA,* we examined five variants, three found within coding exon 5 (CCDS 3769.1) representing two common synonymous SNPs (rs5333 (p.H323H) and rs5334 (p.E335E)) and a missense rare variant, considered a mutation, with no reported MAF (rs17856670 (p.L322V), as well as two rare SNPs (rs112710542 (c.901−50G>A) and rs10305924 (c.1034+19G>A)) located in intron 4 and 5, respectively. Finally, for *NBS1*, we evaluated six variants, five located within coding exon 5 (CCDS 6249.1) including one non-synonymous common SNP (rs1805794 (known as p.E185Q)), a rare synonymous SNP (rs780661058 (p.A183A)), three missense mutations (rs182756889 (p.R169C), rs61754966 (p.I171V), and rs151070415 (p.A183T)) with no reported MAF or global MAF (GMAF) < 0.1%, as well as a rare SNP within intron 5 (rs192240705 (c.584+32T>C)). In the case of *VEGFA* and *FAS,* the (−) and (+) symbols denote nucleotides before and after translation start and end, accordingly, with SNP nomenclature according to the dbSNP database and previous cited literature. For *EDNRA* and *NBS1,* the reference sequences for numbering annotations are NM_001957.3 and NM_002485.5.

### 4.3. Variant Genotyping

Germline DNA was extracted from the peripheral blood samples of patients using a standard desalting method, whereas genotypic analysis was performed by Sanger sequencing, as previously described in detail [19,46]. Concerning *VEGFA*, the primers used for variant detection were (sense and antisense): 5′-AGGATGGGGCTGACTAGGTAA-3′ and 5′-CCCCCTTTTCCTCCAACTCTC-3′ (detects rs699947, rs34376996, rs12664104, rs144854329, and rs35864111); 5′-GCCCATTCCCTCTTTAGCCA-3′ and 5′-AGTGAGGTTAC-GTGCGGACAG-3′ (detects rs833061, rs149983590, and rs833062); 5′-CTGCTCCCTCCTCGCCAATGC-3′ and 5′-CCAAGCCTCCGCGATCCT-3′ (detects rs59260042, rs79469752, rs13207351, rs28357093, and rs1570360); and 5′-TCACCAGGAAAGACTGATACA-3′ and 5′-GGTGGGTGTGTCTACAGGA-3′ (detects rs111933757, rs187429037, rs112005313, rs112256643, rs149179279, and rs3025039). A single primer pair was used for the detection of *EDNRA* variants (5′-ATTCTTTCTCTGGTGTCTGC-3′ and 5′-GAAAATCTGAGAAACTCCAAT-3′); *FAS* variants (5′-TGCGATTTGGCTTAAGTTGT-3′ and 5′-GGCTTCTGCTGTAGTTCAACC-3′) and *NBS1* variants (5′-AGAGAGATGAAAGGGAAA-3′ and 5′-ATTACATCCTGAAACAAGCAT-3′).

All aforementioned primers were M13-coupled for genotyping. This involved PCR amplification on a GeneAmp PCR system, followed by sense and antisense sequencing with M13 forward and reverse primers and the Big Dye Terminator kit v.1.1 (Applied Biosystems, Foster City, CA, USA), according to the manufacturer’s instructions. Samples were purified by ethanol/Ethylene Diamine Tetraacetic Acid (EDTA) precipitation and loaded on an Applied Biosystems (ABI) 3130xl Genetic Analyzer. Sequences were visualized upon capillary electrophoresis and called with the Sequencing Analysis software v5.2 (Applied Biosystems/Biosolutions, Athens, Greece).

### 4.4. Statistical Considerations

Descriptive statistics, including counts with the corresponding percentages (for categorical variables) and medians with the respective ranges (for continuous variables), were used to summarize patient/tumor characteristics and the distribution of the examined variants for the entire cohort by tumor type (NPC vs. non-NPC). The associations of the selected clinicopathological variables with the variants of interest were assessed by the chi-square/Fisher’s exact test and the Wilcoxon rank-sum test.

Overall survival (OS) was defined as the time (in months) from diagnosis to death from any cause. Patients alive and those lost to follow-up were censored at the date of last follow-up. Survival curves were estimated with the Kaplan–Meier product limit method and compared among groups with the log-rank test. The corresponding 95% confidence intervals for the median values were calculated by the complementary log–log transformation. The prognostic significance of variants was estimated by Cox proportional hazard regression analyses under the additive genetic models univariately and after adjustment for already established prognostic covariates including age, stage, and surgery (only for non-NPC patients) for variants that were (marginally) significant univariately. Departures from the proportional hazards assumption for all Cox models was assessed using time-dependent covariates. All tests were two-sided at an alpha 5% level of significance. Analyses were conducted using the SAS (version 9.3) software (SAS Institute Inc., Cary, NC, USA).

## 5. Conclusions

In conclusion, two SNPs in the *FAS* (rs2234768) and *VEGFA* (rs13207351) genes seem to have an association with clinical outcome for patients with HNC, with *VEGFA* rs13207351 being the most promising. These findings need further validation in a bigger cohort of NPC and non-NPC patients for definitive results.

## Figures and Tables

**Table 1:** Patient and tumor characteristics by tumor type.

Parameter	Total (*N* = 333)	Non-NPC (*N* = 189)	NPC (*N* = 144)
Age (*N* = *314*)			
Median (min, max)	60.2 (14.3, 88.3)	64.9 (37.1, 88.3)	51.6 (14.3, 81.5)
	***N* (%)**	***N* (%)**	***N* (%)**
Sex (*N* = *333*)			
Female	52 (15.6)	16 (8.5)	36 (24.8)
Male	282 (84.4)	173 (91.5)	109 (75.2)
Alcohol Abuse (*N* = *292*)			
No	145 (49.7)	47 (26.6)	98 (85.2)
Yes	147 (50.3)	130 (73.4)	17 (14.8)
Smoking (*N* = *293*)			
No	60 (20.5)	19 (10.8)	41 (35.0)
Yes	233 (79.5)	157 (89.2)	76 (65.0)
Histological Type (*N* = *307*)			
Non-Keratinizing Carcinoma	37 (12.1)	0 (0.0)	37 (28.7)
Squamous Cell Carcinoma	189 (61.6)	172 (96.6)	17 (13.2)
Undifferentiated Carcinoma	77 (25.1)	2 (1.1)	75 (58.1)
Other	4 (1.3)	4 (2.2)	0 (0.0)
Primary Tumor Location (*N* = *318*)			
Hypopharynx	1 (0.3)	1 (0.57)	0 (0.0)
Larynx	149 (46.9)	149 (85.6)	0 (0.0)
Major Salivary Glands	2 (0.6)	2 (1.1)	0 (0.0)
Nasopharynx	144 (45.3)	0 (0.0)	144 (100.0)
Oral Cavity	11 (3.5)	11 (6.3)	0 (0.0)
Oropharynx	7 (2.2)	7 (4.0)	0 (0.0)
Paranasal Sinuses	4 (1.3)	4 (2.3)	0 (0.0)
T (*N* = *272*)			
T1	45 (16.5)	19 (11.3)	26 (25.0)
T2	79 (29.0)	34 (20.2)	45 (43.3)
T3	94 (34.6)	77 (45.8)	17 (16.3)
T4	54 (19.9)	38 (22.6)	16 (15.4)
N (*N* = *275*)			
N0	126 (45.8)	114 (67.1)	12 (11.4)
N1	42 (15.3)	14 (8.2)	28 (26.7)
N2	80 (29.1)	33 (19.4)	47 (44.8)
N3	23 (8.4)	5 (2.9)	18 (17.1)
Nx	4 (1.5)	4 (2.4)	0 (0.0)
Stage (*N* = *281*)			
I	19 (6.8)	15 (9.0)	4 (3.5)
II	52 (18.5)	23 (13.8)	29 (25.4)
III	105 (37.4)	61 (36.5)	44 (38.6)
IV	105 (37.4)	68 (40.7)	37 (32.5)
Surgery (*N* = *176*)			
No	33 (18.8)	33 (18.8)	-
Yes	143 (81.3)	143 (81.3)	-
Type of Surgery (*N* = *142*)			
Chordectomy	8 (5.6)	8 (5.6)	-
Hemiglossectomy	5 (3.5)	5 (3.5)	-
Hemiglossectomy and Laryngectomy	2 (1.4)	2 (1.4)	-
Laryngectomy	113 (79.6)	113 (79.6)	-
Other	14 (9.9)	14 (9.9)	-
Treatment Received (*N* = *311*)			
No	44 (14.1)	43 (23.9)	1 (0.8)
Yes	267 (85.9)	137 (76.1)	130 (99.2)
Type of First Treatment Received (*N* = *267*)			
Induction Chemotherapy	10 (3.8)	0 (0.0)	10 (7.7)
Primary concomitant CT and RT	179 (67.0)	65 (47.4)	114 (87.7)
Primary RT	34 (12.7)	33 (24.1)	1 (0.8)
RT after First Progression	1 (0.4)	1 (0.7)	0 (0.0)
Adjuvant	32 (12.0)	29 (21.2)	3 (2.3)
First-Line	11 (4.1)	9 (6.6)	2 (1.5)

The percentages for the type of surgery and the type of primary treatment were calculated from the total number of patients with available data that had undergone surgery and had received treatment, respectively.

**Table 2:** Frequency distribution of the examined variants in the total cohort and by tumor type.

Parameter	Total (*N* = 293)	Non-NPC (*N* = 149)	NPC (*N* = 144)
VEGFA rs699947 (−2578) (*N* = *286*)
A	40 (14.0)	20 (13.4)	20 (14.6)
AC	136 (47.6)	73 (49.0)	63 (46.0)
C	110 (38.5)	56 (37.6)	54 (39.4)
VEGFA rs12664104 (*N* = *285*)			
G	285 (100.0)	149 (100.0)	136 (100.0)
VEGFA rs34376996 (*N* = *258*)
T	258 (100.0)	149 (100.0)	109 (100.0)
VEGFA rs144854329 (*N* = *289*)
het del18bp	148 (51.2)	73 (49.0)	75 (53.6)
hom del18bp	101 (34.9)	56 (37.6)	45 (32.1)
reference	40 (13.8)	20 (13.4)	20 (14.3)
VEGFA rs35864111 (*N* = *289*)
het ins1bp	148 (51.2)	73 (49.0)	75 (53.6)
hom ins1bp	101 (34.9)	56 (37.6)	45 (32.1)
reference	40 (13.8)	20 (13.4)	20 (14.3)
VEGFA rs833061 (-1498) (*N* = *272*)
C	55 (20.2)	31 (20.8)	24 (19.5)
CT	135 (49.6)	75 (50.3)	60 (48.8)
T	82 (30.1)	43 (28.9)	39 (31.7)
VEGFA rs149983590 (*N* = *288*)
C	287 (99.7)	149 (100.0)	138 (99.3)
CA	1 (0.35)	0 (0.0)	1 (0.72)
VEGFA rs833062 (*N* = *276*)			
CT	8 (2.9)	7 (4.7)	1 (0.79)
T	268 (97.1)	142 (95.3)	126 (99.2)
VEGFA rs1570360 (−1154) (*N* = *289*)			
A	91 (31.5)	60 (40.3)	31 (22.1)
G	97 (33.6)	36 (24.2)	61 (43.6)
GA	101 (34.9)	53 (35.6)	48 (34.3)
VEGFA rs28357093 (*N* = *289*)
A	289 (100.0)	149 (100.0)	140 (100.0)
VEGFA rs13207351 (−1190) (*N* = *289*)			
A	136 (47.1)	69 (46.3)	67 (47.9)
AG	64 (22.1)	34 (22.8)	30 (21.4)
G	89 (30.8)	46 (30.9)	43 (30.7)
VEGFA rs79469752 (*N* = *289*)
C	289 (100.0)	149 (100.0)	140 (100.0)
VEGFA rs59260042 (*N* = *289*)
C	289 (100.0)	149 (100.0)	140 (100.0)
VEGFA rs3025039 (+936) (*N* = *285*)
C	200 (70.2)	104 (70.3)	96 (70.1)
CT	71 (24.9)	34 (23.0)	37 (27.0)
T	14 (4.9)	10 (6.8)	4 (2.9)
VEGFA rs149179279 (*N* = *285*)
C	285 (100.0)	148 (100.0)	137 (100.0)
VEGFA rs112256643 (*N* = *285*)
C	285 (100.0)	148 (100.0)	137 (100.0)
VEGFA rs112005313 (*N* = *285*)
G	285 (100.0)	148 (100.0)	137 (100.0)
VEGFA rs187429037 (*N* = *283*)
A	283 (100.0)	148 (100.0)	135 (100.0)
VEGFA rs111933757 (*N* = *280*)
T	280 (100.0)	148 (100.0)	132 (100.0)
EDNRA rs5333 (p.H323H) (*N* = *289*)			
C	13 (4.5)	6 (4.1)	7 (5.0)
CT	114 (39.4)	58 (39.2)	56 (39.7)
T	162 (56.1)	84 (56.8)	78 (55.3)
EDNRA rs5334 (p.E335E) (*N* = *287*)			
A	12 (4.2)	6 (4.1)	6 (4.3)
AG	113 (39.4)	58 (39.2)	55 (39.6)
G	162 (56.4)	84 (56.8)	78 (56.1)
EDNRA rs10305924 (*N* = *285*)
AG	1 (0.35)	0 (0.0)	1 (0.73)
G	284 (99.6)	148 (100.0)	136 (99.3)
EDNRA rs17856670 (p.L322V) (*N* = *289*)
C	289 (100.0)	148 (100.0)	141 (100.0)
EDNRA rs112710542 (*N* = *288*)
AG	6 (2.1)	0 (0.0)	6 (4.3)
G	282 (97.9)	147 (100.0)	135 (95.7)
FAS rs1800682 (-670) (*N* = *290*)
A	74 (25.5)	40 (26.8)	34 (24.1)
AG	165 (56.9)	75 (50.3)	90 (63.8)
G	51 (17.6)	34 (22.8)	17 (12.1)
FAS rs34995925 (*N* = *290*)
T	290 (100.0)	149 (100.0)	141 (100.0)
FAS rs2234768 (−690) (*N* = *290*)
C	1 (0.34)	1 (0.67)	0 (0.0)
CT	52 (17.9)	27 (18.1)	25 (17.7)
T	237 (81.7)	121 (81.2)	116 (82.3)
FAS rs150130637 (*N* = *286*)
A	286 (100.0)	149 (100.0)	137 (100.0)
NBS1 rs1805794 (p.E185Q) (*N* = *291*)
C	37 (12.7)	21 (14.1)	16 (11.3)
G	109 (37.5)	61 (40.9)	48 (33.8)
GC	145 (49.8)	67 (45.0)	78 (54.9)
NBS1 rs192240705 (*N* = *291*)
T	291 (100.0)	149 (100.0)	142 (100.0)
NBS1 rs780661058 (p.A183A) (*N* = *291*)
A	291 (100.0)	149 (100.0)	142 (100.0)
NBS1 rs151070415 (p.A183T) (*N* = *291*)
G	291 (100.0)	149 (100.0)	142 (100.0)
NBS1 rs61754966 (p.I171V) (*N* = *291*)
A	289 (99.3)	148 (99.3)	141 (99.3)
AG	2 (0.7)	1 (0.7)	1 (0.7)
NBS1 rs182756889 (p.R169C) (*N* = *291*)
C	291 (100.0)	149 (100.0)	142 (100.0)

**Table 3:** Hazard ratios and 95% CIs estimated by Cox regression analysis under additive, dominant, and recessive genetic models for VEGFA rs13207351 (−1190) in non-NPC patients.

Parameter	Univariate		*p*-Value	Multivariate *		*p*-Value
	Event/Total	HR (95% CI)		Event/Total	HR (95% CI)	
*VEGFA* rs13207351 (−1190)						
Additive model			**0.012**			**0.031**
A	40/68	2.21 (1.23–3.96)	**0.008**	38/64	2.06 (1.14–3.72)	**0.017**
AG	15/34	1.19 (0.59–2.41)	0.624	14/33	1.19 (0.57–2.45)	0.645
G	16/46	Reference	**1**	16/46	Reference	-
Dominant model						
A+AG	55/102	1.79 (1.02–3.12)	**0.042**	52/97	1.72 (0.98–3.03)	0.059
G	16/46	Reference	**-**	16/46	Reference	-
Recessive model						
A	40/68	2.04 (1.27–3.28)	**0.003**	38/64	1.91 (1.17–3.10)	**0.009**
AG+G	31/80	Reference	**-**	30/79	Reference	-

* Adjusted for age (continuous) and stage (I–II, III–IV). HR, hazard ratio; CI, confidence interval. Significant *p*-values are shown in bold italics.

## Data Availability

The data presented in this study are available upon request from the corresponding author. The data are not publicly available due to ethical and legal issues.

## Supplementary Materials

The following are available online at https://www.mdpi.com/2072-6694/13/5/1163/s1, Table S1: Hazard ratios and 95% CIs estimated by univariate Cox regression analysis under additive genetic models for all examined variants in NPC and non-NPC patients, Figure S1: Analysis of regulatory element markers as well as transcription factor binding sites in loci associated with *VEGFA* rs13207351 from the USCS Genome Browser (http://ge-nome.ucsc.edu, accessed on 15 February 2021) and ENCODE (https://genome.ucsc.edu/cgi-bin/hgTracks?db=hg19&lastVirtMode-Type=default&lastVirtModeExtraState=&virtMode-Type=default&virtMode=0&nonVirtPosi-tion=&position=chr6%3A43737744%2D43737844&hgsid=1030849587_t7VZPWaPfJsrlzTTaw-dln7DOIGoT, accessed on 15 February 2021, Figure S2: Analysis of regulatory element markers as well as transcription factor binding sites in genomic loci associated with *FAS* rs2234768 from the USCS Genome Browser (http://ge-nome.ucsc.edu) and ENCODE (https://genome.ucsc.edu/cgi-bin/hgTracks?db=hg19&lastVirtModeType=default&lastVirtModeExtraState=&virtModeType=default&virtMode=0&nonVirtPosi-tion=&posi-tion=chr10%3A90748194%2D90753813&hgsid=1030946669_QZu9OaqVBNaI1d5rluV4CLcRdd5q, accessed on 15 February 2021).
