# Supplementary material for: Genetic Variation in the Vascular Endothelial Growth Factor (VEGFA) Gene at rs13207351 Is Associated with Overall Survival of Patients with Head and Neck Cancer
Source: Cancers (Basel). 2021 Mar 8;13(5):1163. doi: 10.3390/cancers13051163 (PMC7962814; doi:10.3390/cancers13051163)
Supplement: Supplementary file 1 [file cancers-13-01163-s001.pdf]

cancers

MDPI

Supplementary Materials

Genetic Variation in the *Vascular Endothelial Growth Factor (VEGFA)* Gene at rs13207351 Is Associated with Worse Overall Survival of Patients with Head and Neck Cancer

Foteinos-Ioannis Dimitrakopoulos, Georgia-Angeliki Koliou, Vassiliki Kotoula, Kyriaki Papadopoulou, Konstantinos Markou, Konstantinos Vlachtsis, Nikolaos Angouridakis, Ilias Karasmanis, Angelos Nikolaou, Amanda Psyrri, Anastasios Visvikis, Paris Kosmidis, George Fountzilas and Angelos Koutras

Table S1. Hazard ratios and 95% CIs estimated by univariate Cox regression analysis under additive genetic models for all examined variants in NPC and non-NPC patients (with tumors located in the larynx).

Variant	*NPC Patients*			*non-NPC Patients*		
	**Event/Total**	**HR (95% CI)**	***p*-Value**	**Event/Total**	**HR (95% CI)**	***p*-Value**
**NBS1 rs1805794 (p.E185Q)**			0.605			0.730
C	3/15	0.58 (0.17-2.00)	0.387	10/21	1.33 (0.65-2.73)	0.433
G	13/44	1.10 (0.52-2.32)	0.806	30/60	1.10 (0.67-1.82)	0.703
GC	15/63	Reference	--	31/67	Reference	--
**EDNRA rs5333 (p.H323H)**			0.809			0.728
C	0/5	--	--	4/6	1.33 (0.48-3.73)	0.585
CT	11/46	0.78 (0.38-1.63)	0.515	26/58	0.89 (0.54-1.45)	0.640
T	21/70	Reference	--	41/83	Reference	--
**EDNRA rs5334 (p.E335E)**			0.855			0.728
A	0/4	--	--	4/6	1.33 (0.48-3.73)	0.585
AG	11/46	0.81 (0.39-1.69)	0.575	26/58	0.89 (0.54-1.45)	0.640
G	21/70	Reference	--	41/83	Reference	--
**FAS rs1800682 (-670)**			0.602			0.295
A	9/28	1.22 (0.55-2.68)	0.621	19/40	1.30 (0.74-2.29)	0.366
AG	20/79	Reference	--	32/74	Reference	--
G	2/14	0.56 (0.13-2.40)	0.434	20/34	1.54 (0.88-2.70)	0.128
**FAS rs2234768 (-690)**						0.171
C	--	--	--	1/1	3.97 (0.54-29.19)	0.175
CT	8/20	2.22 (0.98-5.00)	**0.056**	9/27	0.64 (0.32-1.29)	0.215
T	23/101	Reference	--	61/120	Reference	--
**VEGFA rs699947 (-2578)**			0.136			0.890
A	3/18	1.04 (0.27-3.91)	0.959	11/20	1.19 (0.58-2.42)	0.636
AC	18/51	2.17 (0.94-4.99)	0.069	35/72	1.08 (0.65-1.80)	0.774
C	8/48	Reference	--	25/56	Reference	--
**VEGFA rs144854329**			0.182			0.890
het del18bp	21/61	2.03 (0.61-6.81)	0.251	35/72	0.91 (0.46-1.79)	0.780
hom del18bp	7/41	1.00 (0.26-3.86)	0.998	25/56	0.84 (0.41-1.72)	0.636
reference	3/18	Reference	--	11/20	Reference	--
**VEGFA rs35864111**			0.182			0.890

het ins1bp	21/61	2.03 (0.61-6.81)	0.251	35/72	0.91 (0.46-1.79)	0.780
hom ins1bp	7/41	1.00 (0.26-3.86)	0.998	25/56	0.84 (0.41-1.72)	0.636
reference	3/18	Reference	--	11/20	Reference	--
**VEGFA rs833061 (-1498)**			0.731			0.134
C	5/22	1.57 (0.48-5.15)	0.458	12/31	1.20 (0.57-2.54)	0.635
CT	11/47	1.38 (0.51-3.73)	0.526	43/74	1.74 (0.98-3.08)	0.060
T	6/34	Reference	--	16/43	Reference	--
**VEGFA rs1570360 (-1154)**			0.570			0.865
A	5/26	0.79 (0.26-2.36)	0.672	29/60	0.90 (0.53-1.54)	0.703
G	15/55	1.32 (0.58-3.03)	0.512	17/35	1.05 (0.57-1.95)	0.874
GA	9/39	Reference	--	25/53	Reference	--
**VEGFA rs13207351 (-1190)**			0.294			**0.012**
A	14/54	1.85 (0.71-4.82)	0.207	40/68	2.21 (1.23-3.96)	**0.008**
AG	9/27	2.23 (0.79-6.28)	0.127	15/34	1.19 (0.59-2.41)	0.624
G	9/39	Reference	--	16/46	Reference	--
**VEGFA rs3025039 (+936)**			0.826			0.504
C	21/83	Reference	--	48/103	Reference	--
CT	8/30	1.19 (0.53-2.70)	0.670	17/34	1.18 (0.68-2.05)	0.559
T	1/4	0.67 (0.09-4.96)	0.691	6/10	1.61 (0.69-3.77)	0.272

HR, hazard ratio; CI, confidence interval.

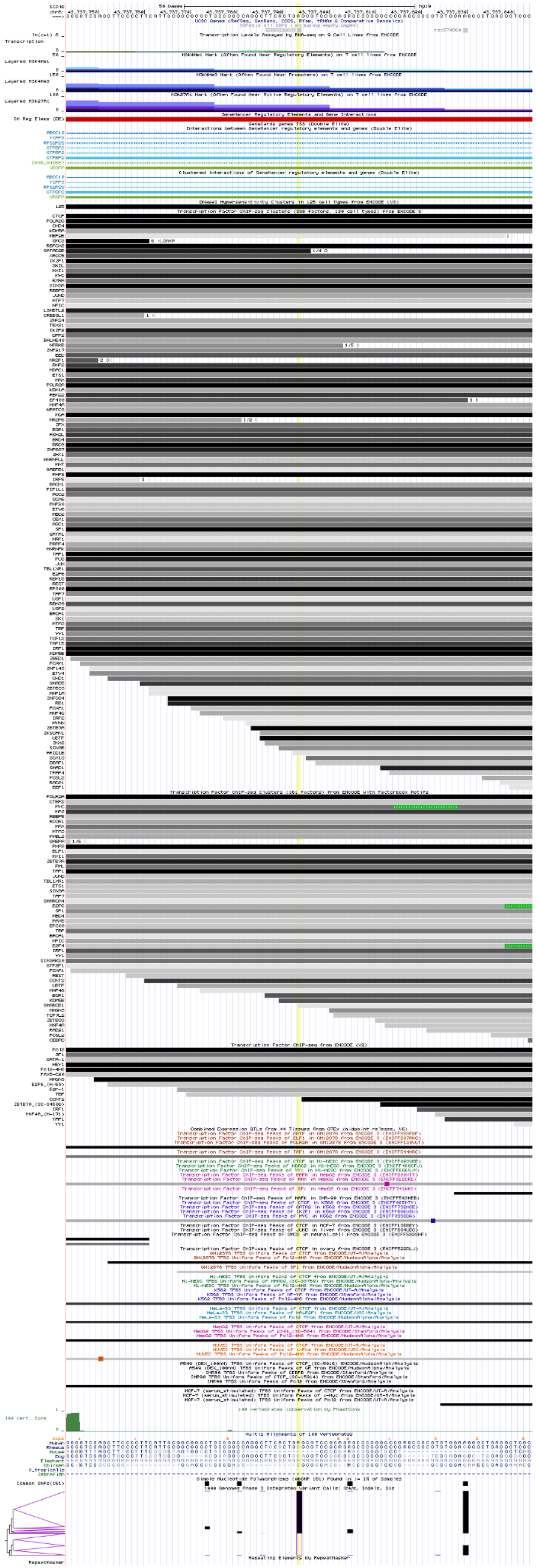

Figure S1. Analysis of regulatory element markers as well as transcription factor binding sites in loci associated with *VEGFA* rs13207351 from the USCS Genome Browser (http://ge-nome.ucsc.edu, accessed on 15 February 2021) and ENCODE (https://ge-nome.ucsc.edu/cgi-bin/hgTracks?db=hg19&lastVirtMode-Type=default&lastVirtModeExtraState=&virtMode-Type=de-fault&virtMode=0&nonVirtPosi-tion=&position=chr6%3A43737744%2D43737844&hgsid=1030849587_t7VZPWaPfJsrlzTTaw-dln7DOIGoT, accessed on 15 February 2021).

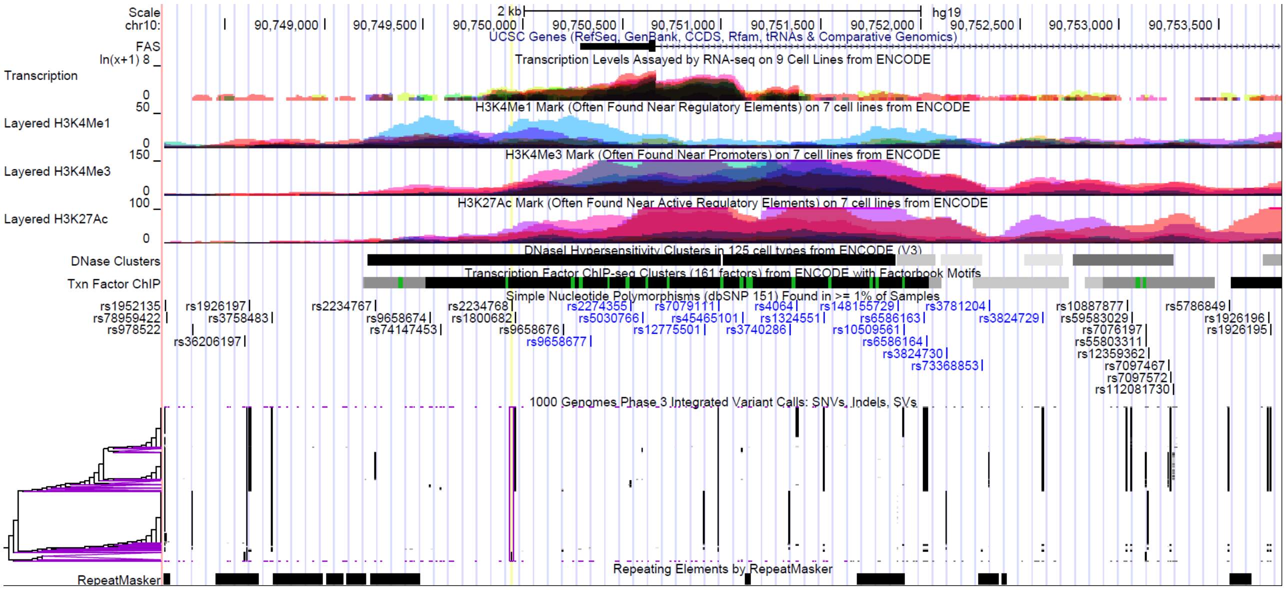

Figure S2. Analysis of regulatory element markers as well as transcription factor binding sites in genomic loci associated with *FAS* rs2234768 from the USCS Genome Browser (http://ge-nome.ucsc.edu) and ENCODE (https://ge-nome.ucsc.edu/cgi-bin/hgTracks?db=hg19&lastVirtModeType=default&lastVirtModeExtraState=&virtModeType=de-fault&virtMode=0&nonVirtPosi-tion=&posi-tion=chr10%3A90748194%2D90753813&hgsid=1030946669_QZu9OaqVBNaI1d5rluV4CLcRdd5q, accessed on 15 February 2021).
